# Interventions and decision-making at the end of life: the effect of establishing the terminal illness situation

**DOI:** 10.1186/s12904-016-0162-z

**Published:** 2016-11-07

**Authors:** C. Campos-Calderón, R. Montoya-Juárez, C. Hueso-Montoro, E. Hernández-López, F. Ojeda-Virto, M. P. García-Caro

**Affiliations:** 1 Foundation of Progress and Health, Andalusian Health Service, Granada, Spain; 2 Nursing Department, University of Granada, Granada, Spain; 3 General Hospital of the Virgen de las Nieves of Granada, Andalusian Health Service, Granada, Spain; 4 Hospital Santa Ana, Andalusian Health Service, Motril, Spain

**Keywords:** Decision making, Delivery of care, End-of-life care, Hospital care, Prognosis, Symptom management

## Abstract

**Background:**

Many ‘routine’ interventions performed in hospital rooms have repercussions for the comfort of the patient, and the decision to perform them should depend on whether the patient is identified as in a terminal phase. The aim of this study is to analyse the health interventions performed and decisions made in the last days of life in patients with advanced oncological and non-oncological illness to ascertain whether identifying the patient’s terminal illness situation has any effect on these decisions.

**Methods:**

Retrospective study of the clinical histories of deceased patients in four hospitals in Granada (Spain) in 2010. Clinical histories corresponding to the last three months of the patient’s life were reviewed.

**Results:**

A total of 202 clinical histories were reviewed, 60 % of which were those of non-oncology patients. Opioid prescriptions (58.4 %), palliative sedation (35.1 %) and Do Not Resuscitate (DNR) orders (34.7 %) were the decisions most often reflected in the histories, and differences in these decisions were found between patients registered as terminal and those who were not registered as terminal. The most frequent interventions in the final 14 days and 48 h were parenteral hydration (96–83 %), peripheral venous catheter (90.1–82 %) and oxygen therapy (81.2–70.5 %). There were statistically significant differences between the patients who were registered as terminal and those not registered as terminal in the number of interventions applied in the final 14 days and 48 h (*p* = 0.01–*p* = 0.00) and in many of the described treatments.

**Conclusion:**

The recognition of a patient’s terminal status in the clinical history conditions the decisions that are made and is generally associated with a lower number of interventions.

## Background

The experience of a patient at end of life is marked not only by the symptoms suffered, but also by other factors, such as the actions and decisions regarding treatment taken by the professionals attending the patient [1, 2]. From a palliative point of view, in the final days, one must consider a decision-making method that has patient comfort as its primary objective and that manages the efficacy and provision of treatments according to this standard.

For physicians, the identification of a patient’s terminal status should represent an inflection point to reflect upon which measures to undertake or not, with the goal of avoiding unnecessary suffering for the patient. However, studies related to end-of-life decisions show the difficulties doctors encounter in recognizing a patient’s proximity to death and/or in noting this proximity in their reports [3–6]. The motives for this difficulty appear to be related to fear of making a mistake [7], the emotional ‘weight’ of the terminal diagnosis [8, 9], and an optimistic attitude regarding the patient’s prognosis [10, 11]. In this same vein, some studies confirm that even in obviously advanced conditions, patients were exposed to life-sustaining interventions and practices, such as mechanical ventilation, transfusions, parenteral nutrition, extrarenal purification, etc., until the end of life [12, 13].

Scientific literature informs the big decisions that direct a more intrusive or a more palliative treatment in a terminal clinical situation. When speaking of end-of-life decision-making, these appear to be centred basically in crucial measures associated with medical aspects, such as sedation, withdrawal of medication, refusal of admission to the intensive care unit (ICU), the omission or withdrawal of antibiotics, hydration, or the use of mechanical ventilation [14, 16]. Similarly, the majority of the studies conducted relate to patients who have died in specialized ICUs [17–19].

However, in a context like the Spanish one, in which the majority of patients in advanced and terminal states are attended in hospital units in the weeks preceding their death, decisions regarding the patient’s comfort relate to interventions that are considered routine.

Interventions such as the insertion of urinary and intravenous catheters, the use of aspirators and other actions that are common for patient care in general hospital units have not been considered in the literature on decision-making in this type of context [20, 21], yet these daily decisions can make the difference between comfort and discomfort for these patients. The recommended best clinical practice for these patients entails limiting the use of actions that will not reduce their suffering and allowing the unrestricted use of interventions that can provide relief, such as providing opioids for pain management and dyspnoea [22–25].

Taking these considerations into account, a descriptive study was designed to review clinical histories to determine the relevant health interventions and decisions performed in the final days of life in hospital rooms. Similarly, the study aimed to determine whether the registration of the patient’s terminal status had any effect on these actions/decisions. Specifically, the objectives of the present study were as follows:

1. To describe the decisions made in the final three months of patients’ lives and the interventions applied 14 days and 48 h before death.
2. To analyse these decisions andcompare the findings for patients registered as being in the terminal stage of illness with those for patients who were not registered as terminal.
3. To compare the decisions made and interventions provided during the final 14 days and 48 h of life in terms of the moment at which the terminal phase was registered.

## Methods

A retrospective study was performed, evaluating the clinical histories of patients who died between January 1 and December 1, 2010 in various public hospitals in the province of Granada (Spain). The study was approved by the ethics committee of each hospital.

### Selection of clinical histories/sample

To calculate the sample size, a total population of 4031 deaths in hospitals and primary care settings was assumed. For a 5 % precision in estimating the proportion using a confidence interval with a normal asymptote with correction for finite populations at 95 % bilaterally, the estimated sample was 252 clinical histories.

Clinical histories were included for patients whose causes of death, as recorded in the death records of the hospitals’ documentation services, were ICD-9 diagnostic codes related to advanced oncological and non-oncological diseases Table 1.

**Table 1:** Data collection protocol

Socio-demographic variables and Clinics	
Sex	
Age	
Cause of death	ICD-9 diagnostics dispatched in the death records of the documentation services of the hospitals included in the study. Oncological disease: 140–195; 196–198; 199; 200–208 Cardiac insufficiency: 402.0.1; 402.11; 402.91, 404; 428; 428.1 Hepatic insufficiency: 571; 572 Respiratory insufficiency/COPD: 491.21; 518.84 Chronic renal insufficiency: 585
Date of death	Dispatched in the death records of the documentation services of the hospitals included in the study
Time elapsed since patient was diagnosed	Noted in the patients’ clinical histories > 1 year ≤ 1 year
Comorbidity according to the Charlson index	Individually calculated for each patient as a function of the diverse pathologies noted in the clinical histories.
Hospital department where patient died	Dispatched in the death records of the documentation services of the hospitals included in the study
Hospitalisation	Number of hospital admissions, final three months of life Number of days of the final hospital admission
Medical end-of-life decisions in the final 3 months of life	
Notation of the decision marked in the clinical history and date at which it first appears. Categorical dichotomy response: Yes-Not noted Affirmative response: Date	- Continuation of care in home - Consult or referral to palliative care - Withdrawal or non-issuance of a determined treatment/intervention. - Decision of withholding/ withdrawing interventions - Not running diagnostics tests - Withdrawal of medication - Withdrawal or non-issuance of antibiotic - Rejection of ICU consult or rejection of ICU ingress - Initiation of opioid medication - Initiation of sedation - Do Not Resuscitate order
Interventions performed in the final 14 days and 48 h	
These interventions were performed at least once in the final 14 days and 48 h of the patient’s life. Categorical dichotomy response: Yes-Not noted.	- Urinary catheter - Central venous catheter - Peripheral venous catheter - Nasogastric tube - Enteral nutrition - Parenteral nutrition - Invasive mechanical ventilation - Non-invasive mechanical ventilation - Transfusion - Aspirator - Aerosols - Oxygen therapy - Antibiotics - Drainage - ICU consult - ICU ingress
Identification of the clinical terminal situation in the final three months of life	
Identification of the terminal situation of the patient and the moment at which this situation is produced. Categorical dichotomy response: Yes-Not noted Affirmative response: Date	Express notation of terminality noted in the clinical histories and date at which the notation is first produced. Other expression(s) used by clinicians to refer to terminality, such as agony, bad prognosis, palliative, and date first produced.

The clinical histories were selected through the centres and were proportional to the number of patients who died in each centre. Deaths that occurred within 24 h following hospital admission were excluded. We also excluded 50 clinical histories of patients who died at home after receiving care from primary care teams because of differences in the format and type of data registered by those teams compared with the data registered by hospitals.

### Procedure

For the selection and identification of clinical histories, the clinical documentation services of each hospital were contacted. Additionally, data for the year 2010 were solicited from the death registries of each hospital where the basic cause of death included any of the aforementioned diseases Table 1.

The clinical histories were selected and reviewed during the year 2011. The documents reviewed in the clinical histories were the registration report and the medical and nursing charts from the last 3 months of the patients’ lives.

Three investigators performed the data collection. To ensure the quality of the data collection and to ascertain agreement in the valuation, a trial was conducted with 12 clinical histories in which certain items that were not easily calibrated were purged and others whose interpretations were unclear were redefined.

Socio-demographic variables were collected, as were clinical data on the diseases. The study variables were the notes in the clinical histories that allowed the identification of the patient’s terminal situation, which was understood as an advanced illness in an evolved and irreversible state with multiple symptoms, emotional impact, loss of autonomy, little to no response to specific treatments and a prognosis of death within weeks or months within a context of progressive frailty [26]. We accepted all annotations that included explicit expressions related to this situation, such as ‘terminal’, ‘agony’, ‘bad prognosis’, and ‘palliative’. Meanwhile, the interventions that the patient received in the final days of life were concretely collected for two time periods: 14 days and 48 h before death. In addition, socio-demographic variables were collected, as were clinical data regarding the diseases.

### Instrument

A data collection protocol was designed that included the aforementioned variables. This document was agreed upon by the investigative group of a broader project called ***‘Variability of Clinical Practice and Conditional Factors in the Implementation of the Processes of Attention at End of Life’***, in which the present work is included Table 1.

### Data analysis

A descriptive analysis of the principle variables studied was conducted. For the quantitative variables, the means, medians, and standard deviations were calculated. The qualitative variables are expressed using absolute and relative frequencies. To represent the data, frequency tables and figures are used.

To ascertain the existence of significant differences among the groups formed using the variables studied (i.e., patients who were registered as being in the terminal stage of illness versus patients who were not registered as such), Student’s *T*-test was used for independent samples, and the Pearson chi-squared test or Fisher’s exact test were used as appropriate for the qualitative variables. For all of the contrasts, a significance level of *p* ≤ 0.05 was considered.

## Results

### Description of the sample

The sample consisted of a total of 202 clinical histories that met the inclusion criteria. The characteristics of the patients’ deaths are shown in Table 2. Regarding the cause of death, 60 % (*n* = 121) of the clinical histories were those of non-oncological patients; of these, 22 % (*n* = 45) were cardiac insufficiency, 20 % (*n* = 40) were respiratory insufficiency/chronic pulmonary obstructive disease (COPD), 11 % (*n* = 22) were hepatic insufficiency and 7 % (*n* = 14) were chronic renal insufficiency. The rest of the clinical histories were of oncological patients (40 %, *n* = 81).

**Table 2:** Characteristics of the patients who were registered as terminal and those who were not

Variables	Total *N* = 202		Registered terminal situation		Registered terminal situation		*p*
			Yes *N* = 104		No *N* = 98		
Age	72.81 years		72.13 years		73.56 years		0.470
	(SD = 13.85)		(SD = 14.69)		(SD = 12.91)		
Sex	Male	Female	Male	Female	Male	Female	0.865
	58.5 % (*n* = 108)	46.5 % (*n* = 94)	52.9 % (*n* = 55)	47.1 % (*n* = 49)	54.1 % (*n* = 53)	45.9 % (*n* = 45)	
Pathology	Oncological	Not oncological	Oncological	Not oncological	Oncological	Not oncological	0.003
	40.1 % (*n* = 81)	59.9 % (*n* = 121)	50 % (*n* = 52)	50 % (*n* = 52)	29.6 % (*n* = 29)	70.4 % (*n* = 69)	
Time elapsed since patient was diagnosed	<1 year	>1 year	<1 year	>1 year	<1 year	>1 year	0.327
	29.7 % (*n* = 46)	70.3 % (*n* = 109)	32.9 % (*n* = 28)	67.1 % (*n* = 57)	25.7 % (*n* = 18)	74.3 % (*n* = 52)	
Comorbidity	Low comorbidity	High comorbidity	Low comorbidity	High comorbidity	Low comorbidity	High comorbidity	0.626
	13.2 % (*n* = 22)	86.8 % (*n* = 145)	14.5 % (*n* = 12)	85.5 % (*n* = 71)	11.9 % (*n* = 10)	88.1 % (*n* = 74)	

*p* ≤ 0.05

Regarding the hospitalization of the patients, 2 % (*n* = 4) of patients were hospitalized at three months, 50.5 % at 14 days (*n* = 102), and 100 % (*n* = 202) at 48 h before death. During the last three months of life, 70 % of patients had one hospital admission, 22.8 % were admitted on two occasions, and 6.4 % were admitted on three occasions. On average, the patients stayed in hospital 13 days (interquartile range 17–22) during their final admission, during which they died.

### Medical end-of-life decisions registered in the clinical histories

**Fig. 1:**
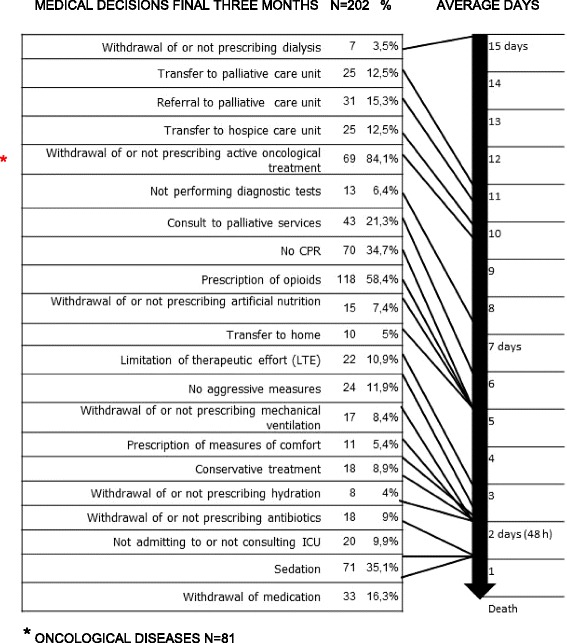
Medical end-of-life decisions registered in clinical histories. * Oncological diseases N=81

Figure 1 shows the number and the total percentage of clinical histories in which the decision was explicitly recorded, along with the average number of days until the moment of death.

The prescription of opioids was the decision most frequently recorded in the clinical histories, with 58.4 % (*n* = 118), followed by palliative sedation with 35.1 % (*n* = 71) and Do Not Resuscitate (DNR) orders with 34.7 % (*n* = 70).

Decisions that were made earlier included the omission or withdrawal of dialysis, with a median of 15 days (SD = 19.84); referral to the palliative care unit, with a median of 10.50 days (SD = 22.91); and transfer to palliative care, with a median of 10 days (SD = 19.65).

Decisions that were made later, at a median of 1 day, were palliative sedation (SD = 6.35), withdrawal of medication (SD = 4.46) and refusal of ICU admission (SD = 9.01).

### Interventions provided during the final 14 days and 48 h of life

**Fig. 2:**
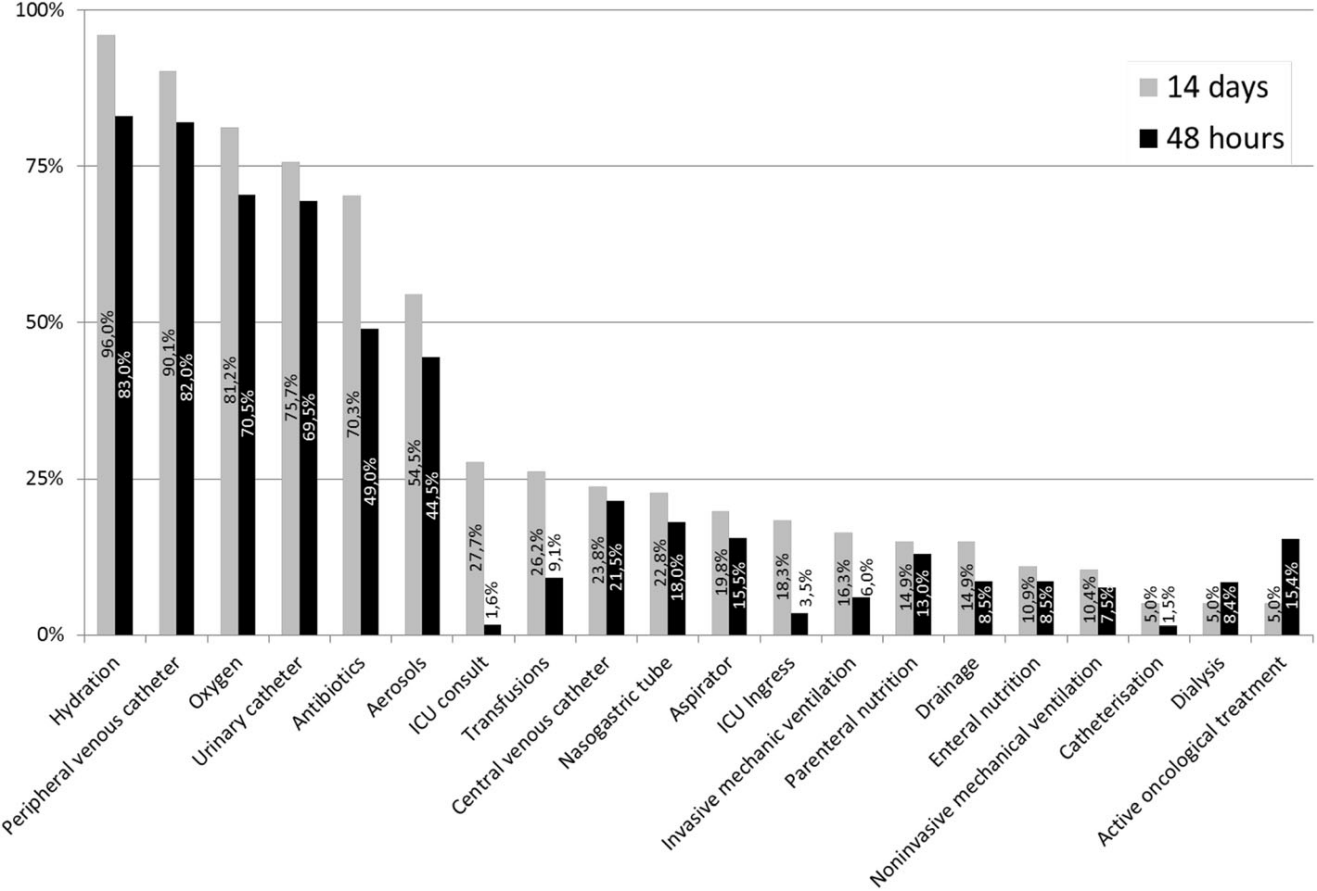
Interventions performed in the final 14 days and 48 h

The interventions documented in the clinical histories in the final 14 days and 48 h of life are shown in Fig. 2.

The most frequently recorded interventions in the final 14 days and 48 h of life, respectively, were parenteral hydration, with 96 % (*n* = 194) and 83 % (*n* = 166); a peripheral venous catheter, with 90.1 % (*n* = 182) and 82 % (*n* = 164); and oxygen, with 81.2 % (*n* = 164) and 70.5 % (*n* = 141).

### Registry of the terminal situation in the clinical histories

In 51.5 % (*n* = 104) of the clinical histories, some language that identified the patient as being in a terminal phase was observed. The median duration between the registration of the patient as terminal and the date of death was 5 days (min 1-max 90). Some 68.27 % (*n* = 71) of these terminal patients were registered before the 48 h immediately preceding their deaths, and the other 31.73 % (*n* = 33) were identified as terminal in the final 48 h.

The group of patients registered as terminal showed characteristics that were similar to the patients who were not registered except for the type of disease, in which significant differences were present between oncology patients and non-oncology patients (*p* = 0.003).

#### Comparison of the decisions related to end-of-life care in patients who were and were not registered as in terminal illness situation

Table 3 shows the frequency of decisions related to end-of-life that were made in the last 14 days of life and were registered in the clinical histories of the patients registered as terminal and those who were not. Significant differences were observed between the two groups regarding decisions to discontinue medications (*p* = 0.000) and to provide DNR orders (*p* = 0.002), palliative sedation (*p* = 0.011) and opioids (*p* = 0.002).

**Table 3:** End-of-life medical decisions made in the final 14 days and 48 h of life for patients who were registered as terminal and those who were not

Medical decisions related to end-of-life care	Decisions in the final 14 days		Decisions in the final 48 h	
	Registered terminal situation Yes	Registered terminal situation No	Registered terminal situation Yes	Registered terminal situation No
	*n* = 104	*n* = 98	*n* = 104	*n* = 98
	n (%)	n (%)	n (%)	n (%)
Transfer to home	5 (4.8 %)	3 (3.1 %)	2 (1.9 %)	0 (0 %)
Transfer to a care home	5 (4.8 %)	8 (8.2 %)	0 (0 %)	1 (1 %)
Palliative consult	14 (13.5 %)	8 (8.2 %)	3 (2.9 %)	1 (1 %)
Referral to palliative care unit	8 (7.7 %)	6(6.1 %)	1 (1 %)	1 (1 %)
Transfer to palliative care unit	9 (8.7 %)	4 (4.1 %)	0 (0 %)	0 (0 %)
Decision of withholding/withdrawing interventions.	12 (11.5 %)	5 (5.1 %)	5 (4.8 %)	3 (3.1 %)
Withdrawal of or not starting artificial nutrition	4 (3.8 %)	1 (1 %)	2 (1.9 %)	0 (0 %)
Withdrawal of or not prescribing parenteral hydration	5 (4.8 %)	0 (0 %)	3 (2.9 %)	0 (0 %)
Withdrawal of or not starting mechanical ventilation	6 (5.8 %)	6(6.1 %)	5 (4.8 %)	5 (5.1 %)
Withdrawal or non-prescription of antibiotics	12 (11.5 %)	1 (1 %)	9 (8.7 %)	1 (1 %)
Not running diagnostic tests	7 (6.7 %)	0 (0 %)	1 (1 %)	0 (0 %)
Withdrawal of medication	29 (27.9 %)	5 (5.1 %)	20 (19.2 %)	5 (5.1 %)
Not entering into or consulting ICU	6 (5.8 %)	5 (5.1 %)	4 (3.8 %)	4 (4.1 %)
Do Not Resuscitate order (DNR)	36 (34.6 %)	15 (15.3 %)	9 (8.7 %)	7 (7.1 %)
Prescription of palliative sedation	38 (36.5 %)	20 (20.4 %)	28 (26.9 %)	15 (15.3 %)
Prescription of opioids	57 (54.8 %)	32 (32.7 %)	21 (20.2 %)	15 (15.3 %)
No aggressive measures	3 (2.9 %)	1 (1 %)	2 (1.9 %)	0 (0 %)
Prescription of comforting measures	14 (13.5 %)	7 (7.1 %)	8 (7.7 %)	4 (4.1 %)
Conservative treatment	3 (2.9 %)	1 (1.0 %)	2 (1.9 %)	0 (0 %)
Withdrawal of or not prescribing active oncological treatment *(oncology patients n = 81)*	3 (5.8 %)	1 (3.4 %)	2 (1.9 %)	0 (0 %)
Withdrawal of or not prescribing dialysis	2 (1.9 %)	0 (0 %)	0 (0 %)	0 (0 %)

Similarly, the frequency of decisions registered in the clinical histories related to end-of-life care was analysed for the last 48 h of life for the patients registered as terminal and those who were not. Comparisons were made between decisions to provide palliative sedation, to prescribe opioids, and to discontinue medication; only the decision to discontinue medication showed significant differences (*p* = 0.002).

Regarding the time points at which decisions were registered in the clinical histories, medical decisions registered in the last 48 h of life were compared for the patients who were registered as terminal before their final 48 h (*n* = 71) and those who were registered later (*n* = 33). Considering palliative sedation, opioid prescription, and the discontinuation of medication, significant differences were only found for opioid prescriptions (*p* = 0.023).

#### Comparison of the interventions performed for patients registered as terminal and those who were not

For the patients who were registered as terminal (*n* = 104), the average number of interventions in the final 14 days was six (min 0-max 22), and the average in the final 48 h was five (min 0-max 14). For the patients who were not registered as terminal (*n* = 98), the average was seven interventions (min 3-max 21) in the final 14 days and six interventions (min 1-max 14) in the final 48 h.

When comparing the number of interventions performed in the final 14 days and 48 h of life between the patients registered as terminal and those who were not, independent of the time point at which this registration was made, we found statistically significant differences for both the final 14 days (*p* = 0.010) and the final 48 h (*p* = 0.001).

The frequencies of the interventions performed in the final 14 days and 48 h of life for the patients registered as terminal and those who were not and a comparison between the two groups are outlined in Table 4.

**Table 4:** Comparison of the interventions performed in the final 14 days and 48 h in patients registered and not registered in a terminal situation

Interventions	Interventions in the final 14 days			Interventions in the final 48 h		
	Registered terminal situation			Registered terminal situation		
	Yes	No	*P*	Yes	No	*P*
	*n* = 104	*n* =98		*n* = 104	*N* = 98	
	n (%)	n (%)		n (%)	n (%)	
Urinary catheter	76 (73.1 %)	77 (78.6 %)	0.363	69 (67 %)	70 (72.2 %)	0.427
Central venous catheter	21 (20.2 %)	27 (27.%6)	0.219	18 (17.5 %)	25 (26 %)	0.142
Peripheral venous catheter	95 (91.3 %)	87 (88.8 %)	0.541	89 (86.4 %)	75 (77.3 %)	0.095
Nasogastric tube	20 (19.2 %)	26 (26.5 %)	0.216	14 (13.6 %)	22 (22.7 %)	0.095
Enteral nutrition	9 (8.7 %)	13 (13.3 %)	0.293	7 (6.8 %)	10 (10.3 %)	---
Parenteral nutrition	13 (12.5 %)	17 (17.3 %)	0.333	10 (9.7 %)	16 (16.5 %)	0.154
Non-invasive mechanical ventilation	5 (4.8 %)	16 (16.3 %)	---	3 (2.9 %)	12 (12.4 %)	---
Invasive mechanical ventilation	7 (6.7 %)	26 (26.5 %)	--	4 (3.9 %)	8 (8.2 %)	---
Transfusions	24 (23.1 %)	29 (29.6 %)	0.293	7 (6.8 %)	11 (11.7 %)	---
Aspirator	15 (14.4 %)	25 (25.5 %)	0.048	10 (9.7 %)	21 (21.6 %)	0.020
Aerosols	50 (48.1 %)	60 (61.2 %)	0.061	38 (36.9 %)	51 (52.6 %)	0.026
Oxygen therapy	83 (79.8 %)	81 (82.7 %)	0.605	68 (66 %)	73 (75.3 %)	0.152
Hydration	99 (95.2 %)	95 (96.9 %)	0.722	81 (78.6 %)	85 (87.6 %)	0.091
Antibiotics	66 (63.5 %)	76 (77.6 %)	0.029	40 (38.8 %)	58 (59.8 %)	0.003
Drainage	17 (16.3 %)	13 (13.3 %)	0.538	10 (9.7 %)	7 (7.2 %)	---
Catheterisation	3 (2.9 %)	7 (7.1 %)	--	1 (1.0 %)	2 (2.1 %)	---
ICU consult	21 (20.2 %)	35 (35.7 %)	0.014	7 (6.7 %)	10 (10.2 %)	---
ICU ingress	12 (11.5 %)	25 (25.5 %)	0.010	8 (7.7 %)	23 (23.7 %)	0.002
Dialysis	5 (4.8 %)	5 (5.1 %)	---	3 (2.9 %)	4 (4.1 %)	--
Active oncological treatment *(in oncology patients n = 81)*	5 (4.8 %)	5 (5.1 %)	--	1 (1 %)	2 (2.1 %)	--

Of the total number of patients registered as terminal (*n* = 104), 68.3 % (*n* = 71) were registered before the final 48 h of life and the rest, 31.7 % (*n* = 33), were registered during the final 48 h. The patients registered as terminal before their final 48 h (*n* = 71) underwent an average of four interventions in their final 48 h, while those who were registered as terminal during their final 48 h (*n* = 33) received an average of six interventions; the difference between the groups is significant (*p* = 0.002). Regarding the differences in the frequency of various interventions between the groups, significant differences were only found for the application of aerosols (*p* = 0.009). In Table 5, the frequency of interventions for both groups is shown.

**Table 5:** Interventions performed for patients registered as terminal with respect to the registration time point

Interventions provided in the Final 48 h	Registered terminal situation before the final 48 h			
	Yes *n* = 71		No *N* = 33	
	*n*	%	*n*	%
Urinary catheter	46	64.8 %	23	69.7 %
Central venous catheter	11	15.5 %	7	21.2 %
Peripheral venous catheter	58	81.7 %	31	93.9 %
Nasogastric tube	8	11.3 %	6	18.1 %
Enteral nutrition	3	4.2 %	4	12.1 %
Parenteral nutrition	6	8.4 %	4	12.1 %
Non-invasive mechanical ventilation	1	1.4 %	3	9 %
Invasive mechanical ventilation	1	1.4	2	6.1 %
Transfusions	2	2.8 %	5	15.1 %
Aspirator	7	9.8 %	3	9 %
Aerosols	20	28.2 %	18	54.5 %
Oxygen therapy	43	60.5 %	25	75.7 %
Hydration	55	77.5 %	26	78.8 %
Antibiotics	23	32.4 %	17	24 %
Drainage	6	8.45 %	4	12.1 %
Catheterisation	0	0 %	1	3 %
ICU consult	3	4.2 %	4	12.1 %
ICU ingress	3	4.2 %	5	15.1 %
Dialysis	2	2.8 %	1	3 %
Active oncological treatment *(in oncology patients n = 81)*	0	0 %	1	3 %

## Discussion

The objective of the present study was to analyse the interventions provided and decisions made in the final days of life and to determine whether registering the patient’s terminal status had an effect on the implementation of these measures. The results appear to show that in the final days of the patient’s life, there is intense therapeutic activity, and this activity is affected by the registration of the terminal phase of the disease in the patient’s clinical history.

In the clinical histories, a great number of ‘routine’ procedures, including parenteral hydration, antibiotics, oxygen, insertion of peripheral venous catheters and urinary catheters, are documented in the final days of the patient’s life. In this sense, our results are consistent with those of other studies centred on patients who died in hospitals during acute disease treatment, in which a greater intensity of interventions were registered at the ends of the patients’ lives [27–29].

Approximately half of the clinical histories include some term that identifies the patient as being in a terminal state, and very few of these clinical histories register this information early. This generalized delay is demonstrated in a considerable number of publications [30, 31].

The identification of the terminal status is related to the decrease in the total number of interventions in the final days of life of the patient. In this sense, the study of Hui et al. [13] shows that the early establishment of the situation of terminal disease in patients with cancer is associated with changes in clinical practice, along with improvements in the quality of care.

According to the results of this study, despite the low frequency of some interventions, we can note that there is not much difference in implementation of interventions such as urinary or nasogastric intubation or central or peripheral intravenous catheterisation between patients who are registered as terminal and those who are not. These results concur with other studies, revealing that even in manifestly terminal conditions, patients were submitted to aggressive measures and important delays in the establishment of palliative objectives [12, 32]. All the same, it is necessary to perform a study with more participants to further support these conclusions.

Measures such as oxygen therapy or hydration deserve special mention, as they can be a clear indication of symptom control. There has been much controversy over hydration and its usefulness at the end of life; however, there is currently sufficient evidence to affirm that hydration does not have positive effects on the patient’s quality of life, nor does it prolong survival [14, 33–36].

Regarding decisions associated with the end of life, we observed that the prescription of opioids and palliative sedation and DNR orders were the most frequently noted.

DNR orders appeared in an important number of clinical histories, both for patients who were registered as being in a terminal illness situation and those who were not. Nonetheless, the proportion found in the present study was lower than in other studies and was documented later in the patient’s illness [13, 27, 37, 38]. Carrion’s [39] study found that a DNR order has an effect on the plan of care; the variations found after signing a DNR order included the withdrawal of treatments and modifications in respiratory support and the administration of vasoactive drugs and dialysis.

Palliative sedation was noted in a high percentage of clinical histories compared with the rest of the end-of-life decisions and was also documented at a later time in the patient’s illness. This finding coincides with the literature, which indicates that sedation is a frequent measure and is often applied late in the patient’s illness [3, 15, 40]. The pressure that family members place on the physician, insistently demanding sedation to alleviate the symptoms and to avoid having to witness their ill family member’s suffering, can condition its implementation [41]. In this sense, some studies indicate that the burden of responsibility that family members sometimes feel regarding the decision to use palliative sedation generates significant feelings of anxiety [42].

It is important to note that medical end-of-life decisions are registered very late in clinical histories. A great number of decisions were documented with a mean time before death equal to or less than 48 h. Any documentation of medical end-of-life decisions with a mean time before death greater than 14 days was almost non-existent.

However, it is interesting to note the great percentage of end-of-life decisions that were documented in clinical histories that did not include an explicit recognition of the terminal status, which suggests that in many cases, formal recognition of the terminal situation in the clinical history is not necessary to produce certain decisions closely related to the end of life.

Regarding the time point at which the terminal situation is registered, it is notable that with the exception of a higher rate of opioid prescription, no differences were observed in the application of interventions or professionals’ decisions in relation to whether the patient’s terminal status was registered in the clinical history.

Regarding limitations, the first to note are those inherent in conducting a retrospective study.

Second, regarding this research, terminal illness situation was considered according only the data reported in the clinical histories reviewed. This means that patients or caregivers were not necessarily aware of this situation at the time of data collection.

Third, the included clinical histories were selected based on cause of death, allowing for a certain number of clinical histories that were not reviewed, as they did not indicate the patient’s underlying disease as the cause of death.

Finally, it is worth noting that the low frequency of implementation of the decisions and interventions that were registered impeded the use of a parametric analysis, which limited the ability to extrapolate some of the results obtained in this study.

## Conclusions

The results of this research highlight that the care received in hospital environments by patients who are in the terminal phase of their disease is characterized by the continuation of numerous interventions and therapeutic decisions that are typical for acute disease and far from the objectives of palliative care. When there is an explicit reference to the terminal phase of a disease in clinical histories, the number of interventions is reduced, and the number of end-of-life-related decisions is increased. However, for many routine interventions, patients continue to be treated like any other patient despite the registration of their terminal situation in the clinical history.

According to these conclusions, it is necessary to undertake without delay educational interventions to improve the training of all professionals of acute care hospitals involved in the care of patients in a terminal illness situation. Secondly, it is also necessary to involve health managers, in the implementation of interventions and procedures to improve management of terminal illness situation in hospital settings, including improving data collected in clinical histories.

## Abbreviations

DNR: Do not resuscitate
ICD-9: International statistical classification of diseases and related health problems (9th version)
ICU: Intensive care unit
